# Liposarcoma: Molecular Genetics and Therapeutics

**DOI:** 10.1155/2011/483154

**Published:** 2010-12-27

**Authors:** Rachel Conyers, Sophie Young, David M. Thomas

**Affiliations:** Sarcoma Genomics & Genetics, Peter MacCallum Cancer Centre, 12 St Andrews Place, East Melbourne, VIC 3002, Australia

## Abstract

Sarcomas are a group of heterogeneous tumours with varying genetic basis. Cytogenetic abnormalities range from distinct genomic rearrangements such as pathognomonic translocation events and common chromosomal amplification or loss, to more complex rearrangements involving multiple chromosomes. The different subtypes of liposarcoma are spread across this spectrum and constitute an interesting tumour type for molecular review. This paper will outline molecular pathogenesis of the three main subtypes of liposarcoma: well-differentiated/dedifferentiated, myxoid/round cell, and pleomorphic liposarcoma. Both the molecular basis and future avenues for therapeutic intervention will be discussed.

## 1. Introduction

An estimated 13,000 people were diagnosed with soft tissue and bone sarcoma in 2009 in America, of which liposarcomas constitute 20% [1, 2]. Despite their rarity these tumours have substantial morbidity and mortality, depending on histological subtype, tumour location, and volume with retroperitoneal sarcomas having particularly poor prognosis [3–9]. Liposarcomas may be classified morphologically into 3 main subtypes consisting of: well-differentiated liposarcoma/de-differentiated liposarcoma (WD/DDLPS), myxoid/round cell liposarcoma (MLPS) and pleomorphic liposarcoma (PLPS) [10]. The morphological diversity of liposarcoma reflects the great variation in biological behaviour ranging from tumours with low metastatic potential, that is, WDLPS, to tumours with high propensity to metastasise, that is, the round cell (RC) variant of MLPS or PLPS [11]. In addition to histological characteristics, anatomical location impacts upon prognosis, given that local control is a prime concern for curative intent. 

Treatment is multimodal with surgical removal and radiotherapy used as cornerstones for local control, along with chemotherapy for systemic disease. Few therapeutic options are available for aggressive local or metastatic disease. Chemotherapy sensitivity varies considerably between subtypes with higher response rates in MLPS compared with WD/DDLPS (48% versus 11%) [12]. MLPS tumours are also highly radiosensitive [13, 14]. Given the small subgroup that is chemo-sensitive, and the overriding lack of chemo-curative disease there are avenues and a need for novel molecular therapies. 

A recent histological and molecular review of 163 liposarcoma and lipomas at the Netherlands Cancer Institute resulted in 23% of tumours being reclassified based on cytogenetic information. This highlights the importance of molecular classification in these tumours and genetic alterations now considered an integral part of the WHO classification [15]. It is hoped that further insight into the molecular characteristics of liposarcomas will allow for accurate subclassification, whilst providing a platform for molecular therapies to be included in the current treatment approach. This paper will outline the current molecular basis of liposarcoma and potential strategies for therapeutic intervention.

## 2. Well- and De-differentiated Liposarcoma

WDLPS represents 40%–45% of all diagnosis of liposarcoma [16]. It is classified as a low-grade neoplasm; it is rarely metastatic and has a low recurrence rate (10%) occurring most often in the retroperitoneum and limbs. The World Health Organization (WHO) classifies WDLPS into three main subtypes: adipocytic, sclerosing, and inflammatory. Adipocytic (lipoma-like) liposarcoma is composed of mature adipocytes, which exhibit variation in cell size and focal nuclear atypia and hyperchromasia [16]. The sclerosing subtype shows scattered distinctive bizarre stromal cells associated with rare multivacuolated lipoblasts set in a fibrillary collagenous background [16]. Finally, the inflammatory subtype shows polyphenotypic lymphoplasmacytic infiltrate, with a B-cell predominance. Less is known about this rare subtype [16–18]. 

DDLPS represents progression from low grade to high-grade nonlipogenic morphology within a WDLPS. DDLPS is more aggressive and exhibits an increased rapidity of disease in contrast to WDLPS, with a metastatic rate of 10%–20% and overall mortality of 50%–75% [4, 7, 19]. In respect to tumour site, retroperitoneal tumours appear to have a worse prognosis [19]. Histologically, DDLPS consists of a WDPLS with a nonlipogenic component, either high-grade, most often resembling malignant fibrous histiocytoma (MFH), or low-grade resembling fibromatosis or low-grade myxofibrosarcoma. The presence of transition from WDLPS to DDLPS is used to differentiate between DDLPS and these other lesions [4, 7, 11, 19–21].

### 2.1. Molecular Genetics

A characteristic feature of WD/DDLPS is the presence of supernumerary ring and/or giant rod chromosomes [22]. These chromosomes contain amplified segments from the 12q13–15 region that can be identified with fluorescence in situ hybridization (FISH) and comparative genomic hybridization (CGH) [23]. Intensive research has identified several oncogenes residing in this region including *MDM2, CDK4, HMGA2, TSPAN31, OS1, OS9, CHOP *and* GLI1* [11, 23–25]. The most compelling evidence to date demonstrates an oncogenic role in WD/DDLPS for *MDM2, CDK4, HMGA2 *and* TSPAN31*. Additional amplification events may also play a role in liposarcoma genesis, for example, c-Jun in the de-differentiation process [26].

**Figure 1:**
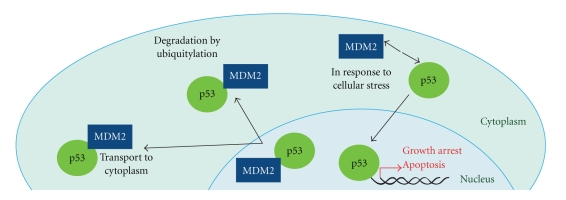
MDM2 binds to the transcriptional activation domain of p53, blocking transcription. MDM2 functions as a ubiquitin ligase, facilitating proteasomal degradation of p53. MDM2 releases p53 in response to cellular stress and p53 translocates to the nucleus where it acts as a transcription factor to enable growth arrest and apoptosis.

*MDM2* amplification is a key feature of WD/DDLPS and is amplified and overexpressed in a number of other cancers, highlighting its importance in tumorigenesis (as reviewed [27]). *MDM2* encodes a negative regulator of the tumour suppressor, p53. MDM2 binds to the transcription activation domain of p53, within an N-terminal hydrophobic pocket [28], blocking p53-dependent transcription [29–33] and recruitment of transcription coactivators [28]. MDM2 also acts as a ubiquitin ligase targeting p53 for proteasomal degradation through both cytoplasmic and nuclear proteasomes [34–36]. MDM2 is involved in its own auto-degradation to prevent MDM2 activity inhibiting p53 during times of cellular stress [37]. Thus MDM2 maintains tight control on cellular p53 levels through multiple mechanisms (see Figure 1) [38, 39]. Therapeutically this is important, as MDM2 inhibitors aim to reactivate p53 and thus allow it to actively induce cell death in response to appropriate stressors [40]. In addition to a functional downstream p53 signalling pathway, MDM2 amplification is a predictor of sensitivity to current MDM2 antagonists [40]. Amplification of *MDM2* and mutation of *p53* appear to be mutually exclusive events in WDLPS, but have been reported in DDLPS [41, 42]. *p53* mutations have been associated with the de-differentiation process from WDLPS to DDLPS [41]. Pilotti et al. reported upon a subgroup of WD/DDLPS tumours. Retroperitoneal WD/DDLPS demonstrate mutual exclusivity between *MDM2* amplification and *p53* mutation. In non-retroperitoneal DDLPS, *p53* mutations occur in the absence of *MDM2* amplification suggesting involvement in the de-differentiation process [41].

**Figure 2:**
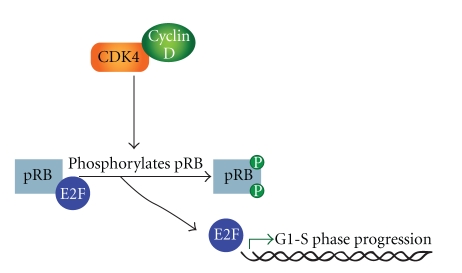
Cyclin dependent kinase CDK4 binds with cyclin D to form active complexes. This results in phosphorylation of Rb and dissociates pRb from the pRb-E2F complex. E2F binds DNA to upregulate transcription of genes required to progress to S phase.

*MDM2* is the most frequent amplification in WD/DDLPS (close to 100%) however *CDK4* is shown to be amplified in over 90% of cases [16, 43, 44]. Given its role in the cell cycle and the frequency of amplification, CDK4 has been well researched in WD/DDLPS. The *CDK4 *gene encodes a 33-kD protein that forms complexes with the cyclin D family, to enable G1-S transition [45]. These CDK4/Cyclin D complexes phosphorylate pRb (encoded by *RB1*), with resultant activation of E2F target genes including E-type cyclins (see Figure 2) [46–48]. It has been suggested that CDK4 provides a selection advantage in WD/DDLPS and may contribute to transformation as CDK4 negative WDLPS exhibit more favorable prognostic features [46]. Coamplification of MDM2 and CDK4 is a common feature of WD/DDLPS and may result in proliferation through combined effects upon p53 and the cell cycle [49, 50]. Interestingly, the rearrangements of chromosome 12 on the giant rod chromosome are discontinuous and *MDM2* and *CDK4* may belong to different amplicons [51, 52]. Several studies [43, 53, 54] have suggested that immunohistochemical staining for both CDK4 and MDM2 may provide a useful diagnostic marker, although FISH and quantitative polymerase chain reaction (qPCR) are more effective. Although MDM2 and CDK4 are useful markers to aid in diagnosis, overexpression of these markers is not unique to WD/DDLPS [43, 54]. Further, the amplification and over-expression of *CDK4* and *MDM2* does not distinguish WDLPS from DDLPS [16, 23, 41]. 

*HMGA2* is similarly located on 12q and frequently amplified in WD/DDLPS. This is a member of the high-mobility group of proteins [55, 56]. Previously referred to as *HMGIC*, it encodes an architectural transcription factor capable of remodeling DNA [57–59]. A direct role for HMGA2 in cellular transformation is demonstrated by NIH3T3 neoplastic transformation with the overexpression of HMGA2 [60]. In human sarcomas during chromosomal rearrangement, *HMGA2* is fused to distant sequences, commonly occurring on other chromosomes and loses its 3′ translated end that also contains sites for *Let-7 *microRNAS [57]. Further support for HMGA2 involvement in adipogenic neoplasm development includes the xenograft model by Arlotta et al. [55] that showed mice expressing C-terminal truncated HMGA2 developed lipomas. Interestingly HMGA2 is frequently coamplified with MDM2 in human malignant tumours [57, 61], particularly WDLPS and DDLPS [52]. This raises the possibility that HMGA2 and MDM2 have a cooperating role in WD/DDLPS. Also included within the chromosome 12 q13–15 region is the transmembrane superfamily gene, sarcoma amplified sequence (*SAS* or *TSPAN31*) gene [62, 63]. *TSPAN31* was originally identified and cloned from an amplified sequence in a malignant fibrous histiocytoma [63]. It has been identified in other subtypes of sarcoma, particularly de-differentiated liposarcoma [64, 65], although its precise role in the de-differentiation process is not well delineated. Forus et al. [66] showed *TSPAN31* was as frequently amplified as *MDM2* in 98 sarcomas. Both TSPAN31 and MDM2 were amplified in 8 of 11 liposarcoma samples, with MDM2 amplified alone in one additional tumour. WDLPS and DDLPS have shown co-amplification of 1q21-q22 and/or 12q21-q22 [11, 16, 23], along with amplification of chromosome 1(1q21-q23). Chromosome 1 amplified sequences include *COAS1*, *COAS2* and *COAS3* [67]. Nilsson et al. showed co-amplification of both *COAS* and *MDM2* in 12/18 lipomatous tumours [68]. The biological function of the *COAS* genes remains a subject for study.

Recent studies into the WDLPS de-differentiation process have suggested a role for the c-Jun N-terminal kinase (JNK) pathway. Co-amplification of 1p32 and 6q23, that contain c-Jun, and Apoptosis Signaling Kinase 1 (ASK1), are seen in DDLPS but not WDLPS [69]. The proto-oncogene *c-Jun* encodes part of the activator protein transcription factor (AP-1) complex involved in cell proliferation, transformation and apoptosis [70]. ASK1 activates JNK [71, 72] ultimately leading to c-Jun activation and PPAR*γ* inactivation. PPAR*γ* is involved in the adipocytic differentiation process and its inhibition may result in de-differentiation. A further role for c-Jun in the de-differentiation process is demonstrated by overexpression in a 3T3-L1 adipocytic tumour xenograft model. Transfection of c-Jun into 3T3-L1 cells *in vitro* delays adipocytic differentiation [26].

## 3. Myxoid Liposarcoma

MLPS is the second most common subtype of liposarcoma and accounts for more than one third of liposarcomas and 10% of all adult soft tissue sarcomas. MLPS is characterized by the presence of spindle or ovoid cells set in a myxoid stroma with signet ring lipoblasts and a distinctive chicken-wire pattern vasculature. The presence of areas with greater cellularity, known as round cell (RC) de-differentiation, is associated with a worse prognosis [73]. Unusual sites of metastasis are common in MLPS with a propensity to metastasize to soft tissue and bone rather than lung [74, 75]. Thirty-one percent of MLPS patients develop metastasis with bone metastases constituting 56% of these [74]. MLPS exhibits inferior survival compared to other low-grade sarcoma subtypes with a 5-year disease survival rate of 85% [76, 77]. MLPS without RC is particularly radiosensitive with good local control rates with patients treated with adjuvant or neoadjuvant radiotherapy approaching 98% 5-year local control [13, 78].

### 3.1. Molecular Genetics

MLPS is characterised by the recurrent translocation t(12;16)(q13;p11) that results in the *FUS-CHOP* gene fusion that is present in over 95% of cases [79, 80]. In most cases, the amino terminal domain of *FUS* (also known as *TLS*) is fused to C/EBP homologous protein (*CHOP*, also known as *DDIT3* or *GADD153*). In rare cases, an alternative translocation event is found t(12;22)(q13;q12) that results in formation of the novel fusion oncogene where *EWS* takes the place of *FUS* [81, 82]. There is strong evidence for these translocations to be the primary oncogenic event in MLPS as these tumours have a relatively normal karyotype, the exception being a few recurrent cases of trisomy 8 [83]. In addition, several growth factor pathways have been implicated in MLPS pathogenesis [84–86].

There are currently 11 different *FUS-CHOP* chimeras and 4 different known *EWS-CHOP* fusion genes. In the most common variants, a portion of the amino terminus of *FUS* is fused to the entire coding region of *CHOP*. The *FUS-CHOP *transcript type does not appear to have a significant impact upon clinical outcome, and RC content, necrosis and p53 expression remain stronger predictors of clinical outcome [79, 87]. There is evidence that the fusion transcript type may influence response to therapy although the studies are hindered by sample size [88–90]. Understanding how the FUS-CHOP fusion causes MLPS and uncovering any further molecular abnormalities in the disease will aid in development of novel targeted therapies.

FUS belongs to the FET family of RNA-binding proteins that consists of FUS, EWS, and TAF15 as well as the closely homologous, Drosophila SARFH (Cab) [80, 91, 92]. These structurally and functionally related RNA-binding proteins are composed of an SYGQ-rich amino terminus, an RNA recognition motif, a zinc finger motif, and at least one RGG rich repeat region [93, 94]. FET proteins are expressed in most human tissues and appear to be regulated following differentiation in neuroblastoma cells and spontaneously differentiating human embryonic stem cells [95].

Both FUS and EWS have been shown to localize to the nucleus and the cytoplasm, bind RNA, and are also involved in nucleo-cytoplasmic shuttling [96–98]. The FET family associate with various complexes involved in the induction of transcription, including RNA polymerase II (RNAPII), which regulates transcription and TFIID complexes, that binds DNA as part of the transcriptional machinery [91], implicating both FUS and EWS in transcriptional control. In addition, FUS has recently been shown to repress transcription of RNA polymerase III (RNAPIII), suggesting a broader role in regulation through multiple different mechanisms [99]. Noncoding RNAs are capable of allosterically modifying FUS in response to DNA damage to inhibit the transcription factor CREB-binding protein (CBP) and p300 histone acetyltransferase activity, resulting in transcriptional inhibition at the cyclin D1 promoter in cell lines and shows a further role for FUS in transcriptional control [100]. FUS has also been implicated in the DNA damage response as a downstream target of ATM, which can detect and coordinate DNA repair [101].

CHOP is induced in response to endoplasmic reticular stress and is involved in mediating cell death in response to such stress stimuli [102]. CHOP also plays a role in regulating differentiation in adipocytes by interfering with the process in response to metabolic stress [103]. Adipocytic differentiation is dependent on the coordinated expression of a group of transcription factors, the CCAAT/enhancer-binding protein (C/EBP) family of proteins [104]. The C/EBP family consists of six members from C/EBP*α* to *ζ*, and they require dimerisation to bind DNA and can form homodimers or heterodimers. CHOP is capable of binding to the C/EBP family members through their highly conserved leucine zipper domain and inhibiting their function. The leucine zipper dimerization domain and the adjacent basic region in CHOP are required for NIH-3T3 transformation with FUS-CHOP, highlighting the requirement for functional DNA binding and dimerization for FUS-CHOP induced oncogenesis [105]. 

As C/EBP*α* and C/EBP*β* play an important role in the adipogenic differentiation and are regulated by CHOP, it is possible FUS-CHOP may interfere in cellular differentiation. In support, various studies suggest that FUS-CHOP functions by inhibiting adipogenesis and maintaining immature adipocytes in a continuous cycle of proliferation without differentiation [106–108]. Introduction of FUS-CHOP into mice, where expression of the transgene is driven by the ubiquitously expressed elongation factor 1*α* (EF1*α*) promoter, results specifically in liposarcomas with inherent induction of adipocyte specific genes such as PPAR*γ* [109]. Further evidence of adipogenic differentiation block resulting from FUS-CHOP expression was shown *in vitro* where mice expressing FUS-CHOP under the control of the aP2 promoter, which is a downstream target of PPAR*γ* expressed in immature adipocytes, failed to develop liposarcomas, indicating interference between PPAR*γ* and aP2 activation [107].

**Figure 3:**
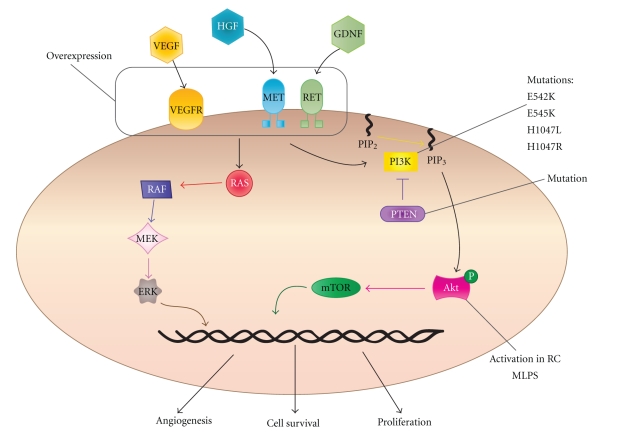
The PI3K pathway is highly active in MLPS, and this is potentiated at least in part by overexpression, and/or activation through RTKs such as MET, RET and VEGFRs. Upon ligand binding, RTKs activate downstream activation of genes involved in multiple cell processes such as cell survival, proliferation, and angiogenesis. These signals are mediated through the PI3K/Akt pathway and also through RAS. PIK3CA and PTEN mutations and Akt activation have also been documented in MLPS.

An emerging clinically relevant targetable pathway in MLPS involves the receptor tyrosine kinases (RTKs) MET, RET, and the PI3K signaling cascade (see Figure 3). RET is overexpressed in MLPS compared to normal fat [84] and high expression has been correlated with poor metastasis free survival in MLPS [108]. RET, IGF1R and IGF2 are highly expressed in MLPS and promote cell survival through both the PI3K/Akt and Ras-Raf-ERK/MAPK pathways [85, 86]. A panel of tyrosine kinases including PDGFRB, EGFR, MET, RET, and VEGFR2 are activated in both treated (with chemotherapy/radiotherapy or Trabectedin) and untreated cases of human MLPS [110]. In addition to activation of MET in clinical MLPS specimens, MET and the ligand HGF are potentially regulated by FUS-CHOP. Both MET and HGF are highly expressed in mesenchymal progenitor cells transfected with FUS-CHOP in a disease mimicking allograft mouse model [111]. In a small clinical cohort, specific Akt phosphorylation was observed in the RC variant and 2 treated cases that harboured *PTEN* mutations, implicating RTK pathways signaling through Akt in MLPS [110]. *FLT1* (that encodes the VEGFR1 protein) is expressed as an indirect downstream effect of FUS-CHOP expression in both FUS-CHOP transfected HT1080 (fibrosarcoma) and MLPS cell lines however, VEGFR tyrosine kinase inhibitors did not have a notable impact on proliferation in MLPS cell lines indicating a separate role in these cells [112, 113]. 

Akt activation, particularly in the RC variant, suggests a role for phosphoinositide 3-kinases (PI3K) [110]. PI3Ks are activated upon phosphorylation of membrane bound receptor tyrosine kinases. PI3K can activate many proteins including the protein serine-threonine kinase Akt, which when phosphorylated causes downstream activation and ultimately cell growth, cell cycle entry, and subsequently survival. The PI3K holoenzyme complex is composed of both a catalytic and regulatory subunit. The catalytic subunit, *PIK3CA*, encodes the p110*α* isoform and is commonly mutated in various cancer types including breast, colon, brain and gastric malignancies [114, 115]. A recent study showed 18% of MLPS patients (*n* = 71) had *PIK3CA* mutations in either the helical (E542K and E545K) or kinase (H1047L and H1047R) domain. The presence of a *PIK3CA *mutation was associated with a shortened disease specific survival [116]. Barretina et al. also showed one tumour with a homozygous *PTEN* mutation. PTEN is a tumour suppressor that dephosphorylates phosphoinositide substrates to negatively regulate the Akt signaling pathway [117], demonstrating more mechanisms for perturbation of the pathway.

## 4. Pleomorphic Liposarcoma

PLPS accounts for only 5% of liposarcomas and occurs mainly within the 55–65 year-old group [8, 118, 119]. PLPS mortality is 40% with no current clinical or pathological predictors of outcome [8, 120]. Histologically PLPS are similar to MFH with the addition of lipoblasts. Histology reveals a disorderly growth pattern, extreme cellularity, and cellular pleomorphism including bizarre giant cells [121]. Lesional cells are polygonal with pale eosinophilic cytoplasm and poorly demarcated boundaries. These lesional cells are interspersed with giant lipoblasts containing enlarged hyperchromatic, angular or globular nuclei [121, 122].

### 4.1. Molecular Genetics

Molecular studies of PLPS are limited by the scarcity of this disease. Tumours tend to show complex arrangements including gains: 1p, 1q21-q32,2q, 3p, 3q, 5p12-p15, 5q, 6p21, 7p, 7q22 (see reviews) [118, 123, 124]. reported literature shows losses i of 1q, 2q, 3p, 4q, 10q, 11q, 12p13, 13q14, 13q21-qter, 13q23-24, (see reviews) [123–125], Taylor et al. described that 60% of PLPS have a deletion of 13q14.2-q14.3, a region that includes the tumour suppressor *RB1* [123]. Also amplified in PLPS, the mitotic arrest deficient (*MAD2*) may also play a critical role [126, 127]. As reported by Singer et al. [126], MAD2 was found to be over-expressed 13 fold in comparison to normal fat, although small sample size (*n* = 6) must be appreciated. As reported by Taylor et al. [123] additional deletions in PLPS include 17p13 and 17q11.2, where *p53* and the sarcoma associated tumour suppressor gene, neurofibromatosis type 1 (*NF-1*) are located. Consistent with these observations, Barretina et al. [116] showed 16.7% of PLPS cases had mutations identified in *p53*, which are rarely seen in MLPS and WD/DDLPS.

## 5. Therapeutic Implications in Liposarcoma

The current modalities available (chemotherapy, surgery and radiotherapy) for the treatment of liposarcoma are limited, creating a need to identify novel therapeutics.

### 5.1. MDM2 Antagonists

Given MDM2 is consistently amplified in WD/DDLPS, and sensitivity to MDM2 antagonists (such as Nutlin-3a) is predicted by MDM2 amplification and an intact wild-type *p53, *it is an appealing therapeutic target [40]. First generation MDM2 inhibitors work via blocking the p53/MDM2 interaction. Nutlin-3a was heralded as one of the most promising MDM2 antagonists when it was shown to activate wild type p53 and induce cell cycle arrest and apoptosis in cancer cell lines [40]. These cell lines included osteosarcoma with amplified MDM2 [40, 128]. Nutlins require wild-type p53 and a functional downstream p53 pathway to be effective [128]. Müller et al. [40] showed downstream p53 dependent transcription and apoptosis in liposarcoma cell lines treated with Nutlin-3a [40]. 

Translation from *in vitro* to attractive *in vivo *therapeutic intervention requires that drugs pass Phase I requirements. Shangary et al. [129] designed spiro-oxindoles as a new class of inhibitors of the MDM2-p53 complex. Spiro-oxindoles bind to MDM2 with high affinity and activates the p53 pathway, inhibiting the growth of neoplastic cell lines with wild-type p53 [129, 130]. MI-219, the lead compound in this class, demonstrates greater potency along with a superior pharmacokinetic profile than Nutlin-3a [129, 131]. MI-219 has been shown to stimulate rapid p53 activation in tumour xenograft tissues with resultant inhibition of cell proliferation [131]. Studies using both Nutlin-3a and MI-219 show a p53 and p21 dependent cell cycle arrest in normal cells, along with p53 dependent cell death specifically in tumour cells [128, 129, 131, 132]. The ability of Nutlin-3a to induce apoptosis in tumours is variable, and osteosarcoma cell lines lacking MDM2 amplification are resistant to apoptosis [131]. Importantly, Nutlin-3a and MI-219 do not cause visible toxicity to animals, as assessed at necropsy [128, 129, 133]. 

Two oral MDM2 inhibitors have recently entered the clinical setting [134], JNJ-26854165 (Ortho Biotech; Johnson & Johnson) [135] and R7112 (Hoffmann-La Roche) [136]. Both agents are available in advanced stage or refractory solid tumours Phase I trials [134]. In addition, AT-219 (a derivative of MI-219) is in preclinical studies with phase I trials planned [134]. Of relevant interest, an MDM2 antagonist RO5045337 is about to recruit for a Phase I trial in liposarcoma patients [137].

### 5.2. CDK4 Antagonists

Targeting CDK4 is an attractive therapeutic strategy given its frequent overexpression in WD/DDLPS [138]. A number of CDK4 inhibitors are in the early pre-clinical development or Phase I and II trials [139]. First generation pan-CDK inhibitors include Flavopiridol and Seleciclib (R-Roscovitine), inhibiting CDK1, CDK2, CDK4, CDK6, CDK7, and CDK1, CDK2, CDK7 and CDK9 respectively [140]. Flavopiridol causes arrest in G1 and G2 phases in a range of solid tumour cell lines [139, 141, 142]. Flavopiridol is more potent if tumour cells are in S phase. Matranga and Shapiro [143] demonstrated recruitment to S phase using hydroxyurea, gemcitabine and cisplatin, followed by flavopiridol resulting in sequence-dependent cytotoxic synergy [143–145]. Flavopiridol and Seliciclib have been investigated in Phase I/II trials for haematological and solid tumours including sarcomas. Trials include Flavopiridol as a single agent and in combination with taxanes where synergism has been noted [141]. Both Flavopiridol and Seleciclib have shown disappointing results relating to clinical outcome and intolerable side effects [146, 147]. 

Newer generation CDK inhibitors include PD0332991, P27600, ZK 304709, R 547 and P1446A05. All are available in Phase I and II solid tumour trials [146]. PD0332991 is one of two more selective CDK inhibitors specific for CDK4 and CDK6. Preclinical data showed inhibition of cell growth through G1 arrest in pRb-positive tumour cell lines and antitumorigenic effects in xenograft models of colon carcinoma [148]. PD0332991 is available in Phase I and Phase II trials for solid and haematological malignancy. Finally, P1446A05 is the only single CDK4 selective inhibitor available [146]. No pre-clinical data is publicly available for this compound; however, it has been released as a Phase I drug for refractory solid tumour and haematological malignancies [146].

### 5.3. PPAR*γ* Ligand Agonists

A critical regulator of terminal differentiation for the adipocytic lineage is a nuclear receptor peroxisome proliferator-activated receptor *γ* [149–151]. PPAR*γ* is an attractive target in undifferentiated lipomatous tumours such as DDLPS and MLPS. PPAR*γ* forms a heterodimeric complex with the retinoid X receptor (RXR). This complex regulates transcription of adipocyte-specific genes by binding sites on DNA. Agonist ligands for the PPAR*γ* receptor have been shown to induce terminal differentiation of normal preadipocytes in human liposarcoma cells *in vitro *[149].

 A Dana-Farber Cancer Institute Phase II clinical trial used Troglitazone, a synthetic PPAR*γ* ligand, in patients with high-grade liposarcoma. This trial enrolled three patients. All patients showed histologic and biochemical differentiation *in vivo*, with reduction in immunohistochemical expression of proliferation marker Ki-67 [149]. A more recent study with 12 patients with Rosiglitazone, belonging to the same class of drugs (thiazolidinediones) as Troglitazone, was not as promising, with median progression free survival of 5.5 months. Treatment did not produce any convincing adipocytic differentiation with no correlation between the high expression of differentiation genes that was found in two patients, and clinical response [152].

### 5.4. Trabectedin (ET-743)

Trabectedin (also known as Ecteinascidin or ET-743) is an antitumor drug isolated from the Caribbean marine tunicate, Ecteinascidia turbinata [153]. Trabectedin is an approved second-line agent for advanced soft tissue sarcoma and has been shown to be exquisitely sensitive to Trabectedin in Phase II clinical trials [154, 155]. The drug is a tetrahydroisoquinoline alkaloid whose main mechanism of action is through binding to the DNA minor groove with promoter and sequence specificity; however, it has also been shown to have effects on promoters that are regulated by major groove binding transcription factors [156–158]. Trabectedin does not appear to effect transcription of FUS-CHOP, but has been shown to dissociate the aberrant transcription factor from promoters of its target genes resulting in removal of the differentiation block by activating a differentiation cascade through the C/EBPs [88]. 

Trabectedin relies on intact nucleotide excision repair (NER) machinery and induces lethal DNA strand breaks in a transcription-couple NER dependant manner [159–161]. It has been suggested that these breaks are repaired by homologous recombination (HR), as HR-deficient cells, such as *BRCA2* mutants, are 100 fold more sensitive to Trabectedin [162]. This effect is specific to HR- mediated double strand break repair as defects in the alternative pathway using nonhomologous end joining do not result in the same degree of Trabectedin sensitivity [161, 162]. 

FUS-CHOP modulates immune genes by activating NF-*κ*B controlled cytokines IL-6 and IL-8 in a C/EBP*β*- dependent manner [163, 164]. Proinflammatory cytokines and growth factors such as CCL2, CXCL8, IL-6, VEGF and PTX3 are highly expressed in both xenograft MLPS models and patient tumours. Trabectedin has been shown to reduce expression and production of these immune modulators, potentially altering the tumour microenvironment in a favorable way [165]. Thus, Trabectedin appears to affect the biological activity of FUS-CHOP and so far shows promise as a therapeutic in MLPS.

### 5.5. Receptor Tyrosine Kinase Pathway Inhibitors

The high frequency of *PIK3CA* and *PTEN* mutations suggests a role for PI3K inhibitors in MLPS. The nonisoform-specific PI3K inhibitors Wortmannin, and LY294002 have been widely used in biological research but are not particularly suited to clinical work due to their lack of specificity, Wortmannin's instability and LY294002's low potency (as reviewed [166]). GDC-0941 and PX-866 are promising PI3K inhibitors currently in clinical trials that have low nanomolar potency against class I isoforms of PI3K [167–169]. In lung cancer cell lines and xenograft models, *PIK3CA* mutants are more sensitive to GDC-0941 [170]. Similarly, *PIK3CA* mutant and PTEN-null tumours were sensitive to PX-866 in xenograft models, and phase I clinical trials for solid tumours are currently underway [169]. The Rapamycin derivate Everolimus inhibits the mTOR complex-1 (mTORC1), which is a downstream effector of PI3K. Both H1047R and E545K PI3K mutant cells are sensitive to Everolimus [171]. PIK3CA mutated MLPS represents an ideal candidate for PI3K inhibition.

As MET is activated in MLPS and there are many MET pathway inhibitors currently in development and in clinical trials (as reviewed in [172], MLPS may be a good candidate for MET inhibition. For example, the novel and promising inhibitor Foretinib (XL880) inhibits multiple kinases including both MET and VEGFR2 and exhibits extensive biological activity and clinical efficacy in an early Phase I clinical trial in metastatic or unresectable solid tumours [173].

## 6. Conclusion

Molecular-based therapeutics are not routinely used in liposarcoma, where surgery, radiotherapy, and chemotherapy remain the mainstay of treatment. Translation of targeted molecular therapeutics in sarcoma has been successfully demonstrated with Imatinib mesylate therapy in c-Kit positive gastrointestinal stromal tumour (GIST) [174]. A major challenge with the use of molecularly targeted therapeutics is to translate disease control into disease eradication. One strategy to achieve this goal is to combine two or more independent molecularly targeted agents in a disease where all of the targets are relevant. The dependence of  WD/DDLPS on amplification of both *MDM2* and *CDK4* means that this disease represents an important candidate for combination therapy. Recent studies point towards RTK involvement in MLPS oncogenesis, particularly signaling through the PI3K/Akt pathway. This provides an important avenue for new research due to the large number of clinical trials currently underway that target this pathway. Although not considered a molecularly targeted therapeutic, treatment of MLPS with Trabectedin is currently in late stage clinical trials with promising results. 

It is hoped that emerging technologies, such as next-generation sequencing, will be fundamental in revealing new molecular targets in liposarcoma. Similarly, advances in drug development should enable improvement of molecular therapies with greater sensitivity, specificity, potency, and limited toxicity. Combining technologies in both areas will allow for efficient clinical translation.
